# Preclinical and clinical evaluation of ECM bioenvelopes for preventing CIED pocket complications

**DOI:** 10.3389/fcvm.2025.1638929

**Published:** 2025-09-12

**Authors:** John N. Catanzaro, Thomas J. Christopher, Ziad F. Issa, Rajasekhar Nekkanti, Huy Phan, Afolabi Sangosanya, Hirad Yarmohammadi, Benjamin D’Souza

**Affiliations:** ^1^ Department of Cardiology, University of Florida Health, Jacksonville, FL, United States; ^2^ Clinical Cardiac Electrophysiology, Sanger Heart & Vascular Institute, Atrium Health, Concord, NC, United States; ^3^ Department of Cardiac Electrophysiology, Prairie Cardiovascular, Springfield, IL, United States; ^4^ East Carolina Heart Institute at ECU Health Medical Center, Greenville, NC, United States; ^5^ Cardiac Electrophysiology, Valley Heart Rhythm Specialists, Chandler, AZ, United States; ^6^ Department of Cardiology, Bay Pines VA Medical Center, Bay Pines, FL, United States; ^7^ Division of Cardiology, Columbia University, New York, NY, United States; ^8^ Department of Cardiology, University of Pennsylvania Medicine, Philadelphia, PA, United States

**Keywords:** CIED, pocket infection, reoperation, biologic envelope, bioenvelope, extracellular matrix, rifampin, minocycline

## Abstract

Cardiac implantable electronic device (CIED) envelopes were developed to secure the device within the surgical pocket, mitigating serious risks for migration or erosion. Available CIED envelopes are either biologic, constructed from non-crosslinked extracellular matrix (ECM), or non-biologic, composed of absorbable synthetic mesh impregnated with antibiotics. Multiple studies have documented constructive remodeling following implantation of the ECM-based bioenvelopes, leading to healthy wound healing and a vascularized surgical pocket. Non-biologic materials, in contrast, trigger a foreign-body response, leading to fibrous encapsulation of the device. Indeed, clinical studies of the bioenvelope have demonstrated constructive remodeling and integration into host tissues. One observational clinical study evaluating CIED reoperations found that patients previously implanted with the bioenvelope had well-vascularized surgical pockets with site-appropriate tissues that facilitated easier device replacement, as opposed to fibrotic encapsulation of the device in patients managed with non-biologic envelopes or without envelopes. A novel, recently approved antibiotic-eluting bioenvelope is designed to provide both support for healthy wound healing plus reduced infection risk, which is a common adverse outcome of CIED implantation. This next-generation bioenvelope includes absorbable discs impregnated with the broad-spectrum antibiotics rifampin and minocycline. Preclinical studies report excellent biocompatibility, biphasic release of antibiotics over 2 weeks, and complete eradication of bacterial inoculates commonly associated with CIED infections. Therefore, this new antibiotic eluting bioenvelope adds standardized drug delivery to the device, without compromising the wound-healing benefits of non-crosslinked ECM.

## 1 Introduction

The use of cardiovascular implantable electronic devices (CIED) continues to grow as the population ages and indications for these devices expand (1, 2). While CIED improve survival and symptom management in appropriate patients, implantation of medical devices poses significant risks, including device migration, erosion, fibrosis, and infection, as well as the challenges of reoperation for device maintenance or upgrade (3). Strategies to mitigate these risks have been developed over recent decades, most notably the use of CIED envelopes to stabilize the devices and multipronged approaches to reducing infection risk.

Erosion or migration of devices are serious adverse outcomes can lead to patient discomfort, device failure (e.g., lead dislodgement), increased infection risk, and reoperation (4, 5). The use of a device envelope can limit these risks by providing the implanting physician with a durable means to secure the device within the surgical pocket. These envelopes are constructed from biologic or non-biologic materials and provide long-term device stabilization through distinct mechanisms. Whereas non-biologic envelopes trigger a robust foreign body response, leading to fibrous encapsulation of the envelope and device, biologic envelopes, constructed from decellularized, non-crosslinked extracellular matrix (ECM), support normal wound healing and constructive remodeling of the material into vascularized tissue. The ECM used in biologic envelopes is composed of non-crosslinked, decellularized porcine small intestinal submucosa (SIS), which contains native structural proteins (e.g., collagen types I and III), glycosaminoglycans, proteoglycans, and signaling molecules that facilitate tissue remodeling. These distinct host responses to biologic and non-biologic envelopes have important implications for wound healing, ease of reoperation, and infection risk.

Pocket infection remains the most common and serious of the potential adverse outcomes associated with implanted devices. Multiple studies have documented the high morbidity, mortality, and financial cost of CIED-related infections (3, 6–9), and have identified risk factors such as patient age, history of prior procedures, type of device, and patient comorbidities (10, 11). Effective strategies to prevent infection are essential to mitigate the substantial burden on individual patients and the overall healthcare system (12, 13).

One evidence-based approach to reducing overall infection risk is the use of an antibiotic-eluting envelope (ABE). For example, the non-biologic ABE (TYRX™, Medtronic, Minneapolis, MN), a synthetic mesh coated with the broad-spectrum antibiotics rifampin and minocycline, has been shown in a randomized trial to significantly reduce the incidence of CIED pocket infections in high-risk patients (14). A biologic ABE that incorporates rifampin and minocycline could have even greater overall clinical benefits. Whereas non-biologic envelopes stimulate a foreign-body response, which can lead to the formation of an avascular, fibrous capsule around the envelope and device, bioenvelopes provide a bioactive substrate that stimulates tissue integration and vascular ingrowth. This support for healthy wound healing minimizes chronic inflammation, foreign body response, and fibrotic encapsulation, and fosters immune access to the tissues surrounding the CIED (15–20).

To combine the benefits of local antibiotic delivery with the regenerative potential of bioenvelopes, a next-generation ABE has been developed and approved for clinical use (EluPro^TM^, Elutia Inc., Silver Spring, MD). The biologic ABE incorporates absorbable, drug-eluting discs impregnated with rifampin and minocycline, which provide high local antibiotic concentrations in the pocket, with minimal systemic exposure. This article presents and reviews clinical and preclinical evidence supporting the utility of biologic envelopes, with a focus on emerging data regarding the potential clinical utility of the novel ABE for stabilizing implantable devices, facilitating reoperation, and minimizing infection risk.

## 2 Design principles of ECM-based bioenvelopes

The clinical benefits of ECM-based biomaterials derive primarily from the ability of intact ECM to influence host response. After the implantation of intact, decellularized ECM, interactions with host immune cells release bioavailable growth factors from the ECM of the biomaterial. These bioactive factors in turn modulate the host immune response, direct the organization of site-appropriate tissues, and stimulate angiogenesis, among other actions (20–25). The immunomodulatory process directed by these bioactive factors further supports long-term pocket health (19, 22, 23, 26). Whereas the immune response to non-biologic materials is characterized by a predominantly M1 macrophage phenotype that is pro-inflammatory and pro-fibrotic, non-crosslinked ECM elicits a predominantly M2 macrophage phenotype, which is anti-inflammatory and promotes immunoregulation and constructive remodeling of tissues (27–32). Furthermore, proangiogenic factors released from within the ECM direct the development of new blood vessels, providing the remodeling bioenvelope with blood supply and therefore greater access to host immune cells. Together, these features of the ECM bioenvelope contribute to the formation of a well-vascularized surgical pocket, as opposed to the fibrous encapsulation triggered by non-biologic materials (15, 20, 21, 33).

As noted, CIED-related infections are relatively common and can greatly increase risks for morbidity, mortality, and additional healthcare costs (9). However, infection risk does not end with the presence or absence of clinical infection following a procedure. Evidence from multiple studies of CIED implantations and reoperations describes a high prevalence of potentially pathogenic bacterial stores persisting in the surgical pocket, even in the absence clinical infection (34, 35). In one study, 33% to 38.5% of asymptomatic patients with CIED at reoperation had evidence of pathogenic bacteria in the surgical pocket (36). Bacteria introduced during implantation or reoperation compete with host tissues for adherence to the surface of the implanted device. Adherent bacteria may form a biofilm, potentially leading to infection when the device is disturbed, such as during reoperation. Device surface characteristics can influence this competition, and it has been suggested that altering the device-host tissue interface may affect bacterial adherence and/or biofilm formation, and therefore potentially reduce infection risk (37). A biologic ABE may convey comprehensive benefits by providing a surface for the adherence and ingrowth of host tissues, promoting vascularization of the tissues around the device, and eluting antibiotics directly into the pocket, thereby minimizing infection risk and facilitating future reoperation.

The CIED bioenvelope is constructed from decellularized porcine small intestine submucosa (SIS) and is soft and conforming in clinical use. Decellularized, non-crosslinked SIS-ECM is a widely used biological scaffold due to its biocompatibility, low immunogenicity, high biological activity, and biodegradability (38). Indeed, SIS-ECM is rich in collagen, proteoglycans, glycoproteins, and growth factors and provides a supportive microenvironment for cell migration, adhesion, and proliferation. Two versions of the bioenvelope, each constructed from 2 perforated multilaminate (4-ply) sheets of non-crosslinked, decellularized, lyophilized SIS-ECM, have received FDA approval: the original SIS-ECM envelope without any antibiotic or antibiotic protocol (CanGaroo®, Elutia, Inc., Silver Spring, MD) and the next-generation biologic ABE, which contains biodegradable discs impregnated with rifampin and minocycline (EluPro^TM^, Elutia Inc., Silver Spring, MD). Aside from the addition of antibiotics, the SIS-ECM biomaterial is identical across both products, each of which has demonstrated consistent regenerative properties in preclinical and clinical studies (33, 39–43). The novel ABE constructed from SIS-ECM is the latest innovation in this product line and is designed to leverage both the long-term regenerative properties of ECM and the direct, local delivery of high antibiotic levels to promote healthy wound healing and minimize infection risk.

## 3 Clinical foundation: the HEAL study

Because of the influence of envelope material (i.e., biologic or non-biologic) on wound healing and encapsulation, the choice of envelope has important implications for long-term device function and ease of reoperation. The need to service or upgrade CIED is a growing issue, as these devices are increasingly used in younger patients with longer life expectancies (1). Generator batteries typically last 6 to 10 years, and younger patients managed with CIED may require several reoperations during their lifetime. In addition, many patients may require reoperation to manage erosion, migration, lead complications, and/or infection. Importantly, each reoperative procedure carries additive risks for adverse outcomes, particularly infection.

Another concern is the potential for fibrotic encapsulation to interfere with device function. Increasing shock impedance and a 20% failure rate with S-ICD was reported by a 2020 study of patients undergoing generator replacement; the investigators hypothesized that fibrotic tissue could contribute to this increasing impedance (44). Based on this hypothesis, a retrospective study of 69 consecutive patients implanted with S-ICD [33 (47.8%) with bioenvelopes] documented impedance levels from the time of implantation to 800 days post-implantation (45). The findings showed an initial decrease in impedance as the pocket healed, followed by a gradual rise in both groups (bioenvelope or no bioenvelope). However, the presence of a bioenvelope significantly attenuated this increase in impedance, an effect that remained statistically significant after application of a multivariate model to account for the non-linear changes in impedance (*P* = .032). The authors suggest that increasing impedance is consistent with the formation of a fibrotic capsule, whereas the attenuation of this increase with ECM bioenvelopes may relate to non-fibrotic (i.e., healthy) wound healing.

Recent evidence from an observation clinical study (HEAL; NCT04645173) indicates that the use of bioenvelopes, through their support for constructive remodeling, lead to reduced fibrous encapsulation and easier mobilization of the device and its leads, thereby reducing procedural difficulty (33). The HEAL study sought to identify differences in implant pockets at reoperation between patients who received bioenvelopes, non-biologic envelopes, or no device envelope during their previous device implantation. Investigators evaluated patient demographics, procedural notes, intraoperative scoring of the pocket, and histology of biopsies from the implant pockets.

### 3.1 HEAL study: patients and materials

The version of bioenvelope used in prior procedures in subjects from the HEAL study consisted of SIS-ECM without any additional antibiotics (CanGaroo®, Elutia Inc., Silver Spring, MD). The non-biologic envelope was constructed from absorbable synthetic mesh composed of glycolide, caprolactone, and trimethylene carbonate polymer, coated with a bioresorbable polyarylate polymer containing rifampin and minocycline. Because the ECM-based bioenvelopes had been shown to facilitate normal pocket healing, it was hypothesized that use of these envelopes during device implantation would facilitate ease of reoperation.

After obtaining IRB approval, investigators evaluated outcomes from 43 patients managed with CIED who required reoperative procedures (11 biologic, 15 non-biologic, 17 no envelope). Subjects were predominantly male (60%), with a mean age of 73.5 years and mean BMI of 30 kg/m^2^ (Table 1). The overall median follow-up was 5.9 years. There were no significant between-group differences in any demographic measure, aside from median time since previous CIED procedure, which was longer in the no-envelope group (9 years vs. 3.9 years for both the biologic and the non-biologic group, *P* < .001).

**Table 1:** HEAL study patient demographics (33). No significant differences were found between groups, except for the number of years since prior implantation which was significantly greater in the no-envelope group.

Patient characteristics	Total	Biologic envelope	Non-biologic envelope	No envelope
Demographics	(*n* = 43)	(*n* = 11)	(*n* = 15)	(*n* = 17)
Age (mean ± SD)	73.5 ± 14.0	66.2 ± 21.0	78.1 ± 6.6	74.2 ± 12.0
Age (min-max)	29–91	29–89	63–88	48–91
Gender, male	26 (60%)	7 (64%)	10 (67%)	9 (53%)
Race, white (*n*, %)	36 (84%)	8 (73%)	13 (87%)	15 (88%)
Ethnicity, Non-Hispanic or Latino (*n*, %)	42 (98%)	11 (100%)	15 (100%)	16 (94%)
BMI, kg/m^2^ (mean ± SD)	30.0 ± 5.4	27.9 ± 4.0	29.2 ± 5.7	32.1 ± 5.4
Prior implant procedures	(*n* = 43)	(*n* = 11)	(*n* = 15)	(*n* = 17)
Left pectoral location (*n*, %)	39 (91%)	8 (73%)	15 (100%)	16 (94%)
≥ 2 leads (*n*, %)	37 (86%)	7 (64%)	14 (93%)	16 (94%)
# Years implanted (mean ± SD)	5.9 ± 3.7	3.9 ± 2.3	3.9 ± 2.4	9.0 ± 3.4*
# Years implanted (min-max)	0.7–15.1	0.7–8.6	0.7–7.6	0.9–15.1

* *P* < .001 vs. biologic or non-biologic envelope groups.

### 3.2 HEAL study results

Overall, reoperations were considered less challenging on patients who received bioenvelopes during their prior procedure (Table 2). Using a 10-point scale, implanting physicians scored reoperations on patients in the biologic group to be significantly easier compared to the no-envelope group [46% easier for generator mobilization [*P* = .02], 41% easier for lead mobilization [*P* = .01], overall 43% less difficult [*P* = .04]]. A similar trend emerged when comparing the biologic and non-biologic groups [39% easier generator mobilization [*P* = .06]; 23%, easier lead mobilization [*P* = .28]; 33% overall less difficult [*P* = .15]]. Scores for lead adhesion classification were also significantly less severe in the biologic group compared to the no-envelope group (*P* = .003), and significantly fewer capsulectomy procedures were required in the biologic compared to the non-biologic group (83% fewer, *P* = .04), and numerically fewer were required compared to the no-envelope group (78% fewer, *P* = .10).

**Table 2:** HEAL study results: evaluation of the reoperative pocket (33).

Subjective measures	Biologic envelope *n* = 11	Non-biologic envelope *n* = 15	No envelope *n* = 17
Lead adhesion classification, mean (range)	1 (0–2)*	1 (1–2)	2 (1–3)*
Capsulectomy required, *n* (%)	1 (9)	8 (53)	7 (41)
Generator mobilization score, mean ± SD	2.0 ± 1.3*	3.3 ± 2.2	3.8 ± 2.2*
Lead mobilization score, mean ± SD	3.0 ± 2.0*	3.9 ± 2.2	5.1 ± 2.1*
Overall procedural difficulty, mean ± SD	2.4 ± 2.1*	3.6 ± 2.1	4.2 ± 2.1*
Objective measures	*n* = 6	*n* = 12	*n* = 14
Capsule thickness, mm (mean ± SD)	0.4 ± 0.1	0.6 ± 0.3	0.6 ± 0.2

* Denotes *P* < .05 between biologic and non-biologic and/or no-envelope groups.

**Figure 1:**
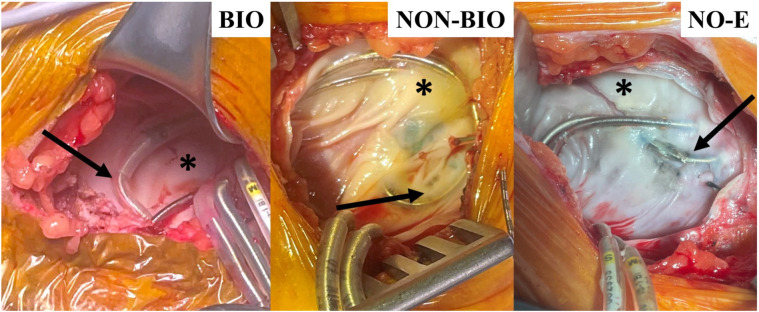
Characteristic photographs of cohort reoperative pockets in the HEAL study (33). Arrows show lead entrapment within the capsule for non-biologic (NON-BIO) and no-envelope (NO-E) groups compared to none in the biologic (BIO) group. Asterisks show areas of thick fibrous tissue in the NON-BIO and NO-E capsules compared to thinner, vascularized tissue in the BIO group.

These outcomes are illustrated by the representative images in Figure 1, which shows that device leads were often entrapped in fibrous tissues in the non-biologic and no-envelope groups, and these groups tended to have thicker, more fibrous capsules, compared to the well vascularized tissues around devices in the biologic group. Indeed, independent, blinded histologic evaluation of pocket biopsies (6 biologic, 12 non-biologic, 14 no-envelope with complete datasets) showed 30% thinner capsules in the biologic implant pockets compared to the no-envelope group (*P* = 0.12), and 32% thinner capsules compared to the non-biologic group (*P* = .09).

Overall, the HEAL study provided insights into the long-term clinical benefits of the bioenvelope when used in CIED implantations. The use of bioenvelope led to fewer lead adhesions, easier generator and lead mobilization, thinner tissue capsules, and reduced need for capsulectomy during reoperation. Similar findings have been reported in preclinical studies and case reports. A recent study in a rabbit model, which compared implantation of the bioenvelope (without antibiotics) plus a pacemaker to insertion of a pacemaker alone, reported a 5-fold reduction in device movement with use of the bioenvelope (46). The bioenvelopes showed progressive resorption and revascularization during the 26-week study period. Thus, the bioenvelope stabilizes the CIED within the pocket through the development of vascularized tissue surrounding the device, which, as suggested by the HEAL study, may facilitate device removal when future exchange or revision is required (47, 48). A recent case report further indicated that placing a bioenvelope within an existing fibrotic capsule during reoperation can in fact foster the development of new non-fibrotic tissue within the pocket, suggesting that the bioenvelope may allow for the reuse and rehabilitation of existing fibrotic implant pockets (49).

## 4 Proof of concept: gentamycin-soaked bioenvelope as an antibiotic-eluting system

During the implantation of CIEDs, microorganisms may be introduced onto tissues surrounding the surgical site and/or the device surface and may persist in the pocket, despite the absence of signs or symptoms of clinical infection (34, 35). These latent bacterial stores exist in a delicate balance with host immune factors and may be released following disturbances, such as reoperation, thereby increasing the risk of subsequent infection (50, 51). Because all infections begin with bacterial colonization (6, 52), evidence-based strategies to reduce bacterial burden include chlorhexidine skin preparation, preoperative antibiotics, meticulous surgical technique, and the local administration of antibiotics into the pocket (13, 14, 53–55). The additional use of ABE is supported by accumulating evidence of their efficacy for further reducing infection risk (14, 56, 57).

To compliment the regenerative properties of ECM with antibiotic elution, researchers developed a protocol to soak the bioenvelope in gentamycin preoperatively. This strategy was based on the common practice by implanting physicians of soaking dehydrated bioenvelopes in antibiotic solutions prior to inserting the CIED. One large observational study (*N* = 1,102 patients receiving CIEDs), for example, found that physicians demonstrated a preference for bioenvelopes soaked in solutions containing gentamycin for higher risk cases, a strategy that was associated with a 3-fold reduction in infection risk on multivariate analysis, when controlling for obesity and diabetes (58). Further evidence comes from a retrospective study of consecutive patients implanted by a single physician, which found no differences in infection rates between those who received biologic ABE (soaked in vancomycin and gentamycin) or non-biologic ABE (impregnated with rifampin and minocycline). It is worth noting that patients managed with antibiotic-soaked bioenvelopes in this study, on average, had a greater number of infection risk factors –such as heart failure, use of systemic anticoagulants, advanced age– and were more likely to be undergoing reoperation, suggesting that the implanting physician preferred biologic ABE for higher risk patients (57).

The efficacy of the gentamycin-soaked bioenvelope was established in preclinical studies (39, 40). *in vitro* time-kill experiments demonstrated rapid reductions in bacterial colonies when the ABE was placed in bacterial cultures, with near or complete bacterial eradication within 6 hours (39). The envelopes eluted high gentamycin concentrations in a biphasic pattern, with an initial bolus of drug elution followed by more gradual and sustained release over 1–2 weeks (40). Following implantation of gentamycin-soaked devices into animals inoculated with common CIED pathogens, dramatic (≥3-log) reductions occurred for all tested bacteria, with complete eradication in most experiments (39).

While initial evidence suggests that the approach of soaking the bioenvelope in gentamycin is a safe and effective way of providing high and efficacious local gentamycin levels to the surgical pocket, it is difficult to standardize. Antibiotic efficacy and safety could vary based on drug loading variables, such as the amount and concentration of gentamycin solution, time soaking the bioenvelope in antibiotic solution, and the amount of drug absorbed by individual bioenvelopes.

## 5 Development and preclinical characterization of a bioenvelope with antibiotic-eluting discs

**Figure 2:**
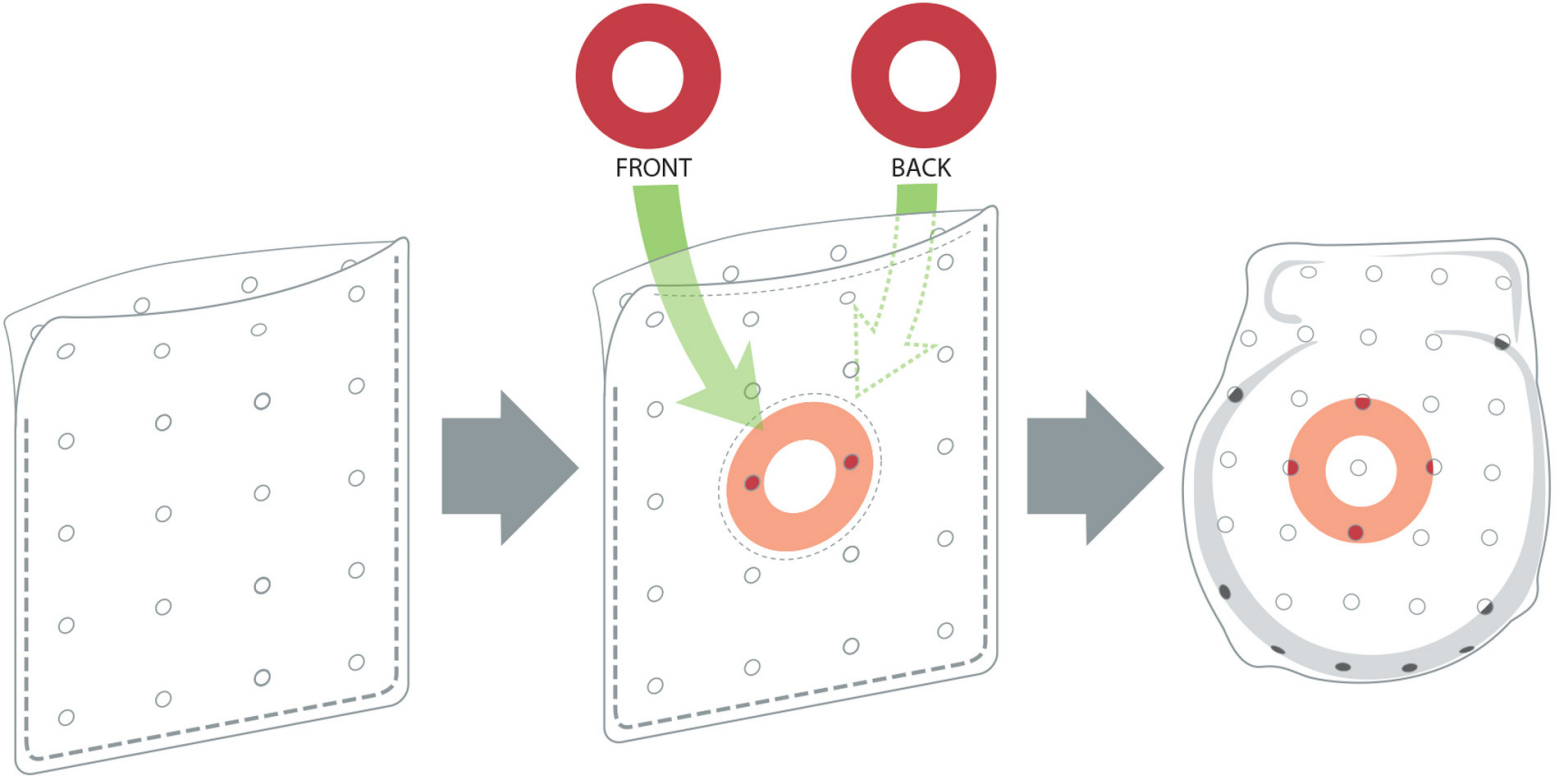
Images of the next-generation antibiotic-eluting bioenvelope, showing the original bioenvelope (left), with PLGA antibiotic-eluting discs (center) and placement of a CIED in the bioenvelope (right).

To overcome the limitations of the soak-in method, a novel ABE was developed by adding drug-eluting discs to the SIS-ECM. The design of this antibiotic-eluting bioenvelope is intended to preserve the bioactive properties of the ECM, while providing high levels of broad-spectrum antibiotics to the surgical pocket. Rather than using a traditional drug coating or impregnation technology, antibiotic-eluting bioenvelope incorporates drug-eluting discs made from a bioabsorbable polymer. This approach minimizes any physical or chemical impacts on the ECM, thereby preserving the surface properties and porosity of the material that are critical to the promotion of cell infiltration and proliferation. Absorbable ring-shaped discs of Poly(D,L-lactide-*co*-glycolide) (PLGA, 50:50) containing rifampin and minocycline are immobilized between the multilaminate SIS-ECM sheets on each side of the bioenvelope, using a circular stitching pattern of 5-0 polydioxanone sutures in the center of the ring (Figure 2). The ABE is designed to provide biphasic antibiotic release, with a high initial bolus followed by gradual elution over approximately 2 weeks. This antibiotic-eluting bioenvelope was cleared by the FDA for clinical use in 2024.

The clinical efficacy of rifampin and minocycline for reducing surgical site infections has been well-established for both CIED-related infections and infections associated with other medical devices (14, 59–61). Indeed, currently available non-biologic ABE, which have been shown to reduce CIED-related infections in a randomized study, leverage the combination of rifampin and minocycline. Together, these antibiotics provide broad-spectrum coverage for the most common CIED-related pathogens, including the gram-positive species cultured from ∼90% of all CIED infections [e.g., *Staphylococcus aureus, S epidermidis,* methicillin-resistant *S. aureus* (MRSA)], as well as Gram-negative species found in some CIED infections (e.g., *Escherichia coli, Acinetobacter baumannii, Haemophilis influenzae*) (62–67).

Initial preclinical studies of this novel ABE support its safety and biocompatibility, as demonstrated through a comprehensive series of ISO 10993-compliant evaluations, which are required by the FDA for device clearance (Table 3) (68). Cytotoxicity testing (ISO 10993-5) confirmed no harmful effects on cells. Sensitization (ISO 10993-10) and irritation (ISO 10993-23) studies showed no signs of allergic response, erythema, or edema. Pyrogenicity testing (ISO 10993-11) revealed no fever response following intravenous injection of extracts. Systemic toxicity testing in mice showed no adverse effects after administration of the material. Long-term implantation studies (ISO 10993-6 and −11) confirmed the absence of local or systemic reactions for up to 26 weeks. These data align with the results of studies of earlier versions of the bioenvelope, which reported no toxic effects, hypersensitivity, or other findings suggesting poor biocompatibility of the device.

**Table 3:** Summary of biocompatibility testing for antibiotic-eluting bioenvelope with rifampin and minocycline (68).

Test category	ISO standard	Model	Duration	Key findings
Cytotoxicity	ISO 10993-5	*in vitro* (cell)	72 h	No cytotoxic effects observed; robust test confirmed safety.
Sensitization	ISO 10993-10	Guinea pig	N/A	No evidence of delayed-type hypersensitivity after intradermal injection.
Irritation	ISO 10993-23	Rabbit	N/A	No erythema or edema; no signs of local irritation after intradermal injection.
Pyrogenicity	ISO 10993-11	Rabbit	N/A	No pyrogenic response; extracts injected intravenously and temperature monitored.
Systemic toxicity	ISO 10993-11	Mice	72 h	No systemic toxicity observed; extracts tested via IV and IP injections.
Systemic & local effects	ISO 10993-6/11	Rabbit	13 & 26 weeks	No adverse systemic or local effects observed; bioenvelope with dummy CIED tested.

The new biologic ABE provides multiple mechanisms to reduce infection risk. Most obviously, the ABE elutes high levels of rifampin and minocycline into the surgical pocket, with strong antimicrobial efficacy and prevention of infection in recent preclinical assessments (41–43). In addition, as described above, the intact ECM of the bioenvelope also supports neovascularization, which provides host immune access to the remodeling tissues, and has been shown to release peptides with direct antibacterial activity (20, 23–25).

The antibacterial efficacy of the antibiotic-eluting bioenvelope was evaluated in a New Zealand White rabbit model, in which either the antibiotic-eluting bioenvelope or a control material (bioenvelope without antibiotics) was implanted in a dorsal subcutaneous pocket (41). Each pocket was then injected with 1 of 4 strains of bacteria commonly found in CIED infections. During the 7-day follow-up, animals that received the antibiotic-eluting bioenvelope had significantly improved health outcomes, with no signs of infection or abnormal body temperatures. This finding contrasts with the no-antibiotic control group, in which several animals developed hyperthermia (3 of 20) and multiple required supportive care (7 of 20). In the antibiotic-eluting bioenvelope group, complete eradication of bacterial colonies and greater than 6-log reductions in colonization were demonstrated for all bacterial strains (Table 4). Post-necropsy macroscopic examination confirmed the absence of infection at the implant sites of animals receiving the biologic ABE. Pharmacokinetic analysis showed sustained local antibiotic concentrations at the implant site for up to 14 days, with minimal systemic exposure, demonstrating the advantages of localized drug delivery.

**Table 4:** Antibiotic-eluting bioenvelope performance against bacterial species in a rabbit model (41).

Organism	Gram stain	Inoculum	Inoculum recovery (mean CFU) *n* = 10	Antibacterial efficacy	Reduction of bacterial colonization
*S. epidermidis*	Positive	10^8^	CIED: 0	> 8-log	Complete kill
			Host tissue: 0		
			Biologic ABE: 0		
*MRSA*	Positive	10^6^	CIED: 0	> 6-log	Complete kill
			Host tissue: 0		
			Biologic ABE: 0		
*A. baumannii*	Negative	10^6^	CIED: 0	> 6-log	Complete kill
			Host tissue: 0		
			Biologic ABE: 0		
*H. influenzae*	Negative	10^6^	CIED: 0	> 6-log	Complete kill
			Host tissue: 0		
			Biologic ABE: 0		

A second recent study further demonstrated that the antibiotic-eluting bioenvelope exhibits a biphasic antibiotic elution profile and maintains broad-spectrum antibacterial activity against seven clinically relevant Gram-positive and Gram-negative organisms commonly implicated in CIED infections (42). Antibacterial efficacy was confirmed using an assay originally developed by the American Association of Textile Chemists and Colorists (AATCC Test Method 100), which quantifies bacterial reduction through colony-forming unit (CFU) counts. In this study, the method was adapted to simulate post-implantation conditions at 2 weeks *in vivo*—an important timepoint when antibiotic concentrations are diminished but still reflect the sustained, extended release of both drugs. The antibiotic-eluting bioenvelope consistently prevented bacterial colonization, even when challenged with high bacterial loads ranging from 10^5^ to 10^7^ CFU, and eradicated all tested organisms. This high level of antibacterial activity was maintained throughout a 12-month product aging period to ensure durability of efficacy over a long product shelf-life. This modified approach provided a more rigorous and clinically relevant evaluation of the antibiotic-eluting bioenvelope's performance, demonstrating its ability to maintain potent antimicrobial activity under physiologic conditions even after partial drug depletion within the matrix.

Complementing these findings, a novel accelerated *in vitro* elution (IVE) method was developed and validated to assess the quality and consistency of the antibiotic-eluting bioenvelope (43). Designed to simulate long-term *in vivo* exposure under *in vitro* conditions, the method enabled rapid evaluation of antibiotic release kinetics. Importantly, it addressed key limitations of the antibiotic soak method described above by providing a standardized, reproducible approach to assess sustained drug release. The IVE method proved essential for demonstrating lot-to-lot consistency during manufacturing and confirmed the reliable and extended elution of rifampin and minocycline over clinically relevant timeframes. These studies support the robustness of the antibiotic-eluting bioenvelope and its potential to prevent infection during the critical early post-implantation period.

In addition to the local provision of broad-spectrum antibiotics, antibiotic-eluting bioenvelope mitigate infection risk through direct and indirect mechanisms inherent to intact ECM. As discussed, the ECM of the bioenvelope supports neovascularization and the development of site-appropriate tissues. In contrast to the avascular fibrotic encapsulation associated with non-biologic devices, the revascularization of the pocket tissues after implantation of a bioenvelope provides immune access to the tissues immediately surrounding the device, where bacterial colonization often occurs (23). While the primary function of an antibiotic-eluting bioenvelope is to prevent local surgical site infections at the CIED pocket, this local protection may also play a critical role in preventing progression to systemic infection. Pocket infections are often the initial source of bacteremia and lead infections, which can disseminate and require more aggressive interventions such as transvenous lead extraction (TLE). Studies have shown that patients who undergo TLE for infective indications, particularly systemic infections, experience significantly higher rates of complications and mortality compared to those undergoing TLE for non-infective causes (69). This highlights the importance of early intervention at the local level to mitigate the risk of systemic spread. Furthermore, elderly patients who develop systemic CIED infections have been shown to suffer from higher long-term mortality following TLE, underscoring the vulnerability of this population and the need for effective infection prevention strategies at the time of device implantation (70). By delivering high local concentrations of antibiotics directly to the pocket and supporting favorable tissue remodeling, biologic ABEs may reduce the risk of bacterial colonization, prevent the cascade of events leading to systemic infection, and ultimately lessen the need for complex and high-risk extraction procedures.

Reoperations are a reality of clinical care for patients with CIED. Many patients, particularly younger patients, will require multiple reoperations for generator replacements or other device maintenance. Furthermore, infection and reoperation are highly interrelated. An increased number of prior CIED-related procedures has been correlated with increased infection risk, and CIED infection is itself considered an indication for reoperation and replacement (10, 11, 69). Although practice patterns vary, large-scale studies of the management of CIED-related infections suggest that device extraction is associated with lower mortality, particularly if performed within days after a diagnosis of infection (69). The HEAL study illustrated the advantages of bioenvelopes in the setting of reoperation, which include ABE: Clinical Advantages.

Taken together, studies of the antibiotic-eluting bioenvelope indicate that the ECM of the bioenvelope is a suitable and effective material for delivering antibiotics to the surgical pocket. Advantages of the antibiotic-eluting bioenvelope with absorbable discs over antibiotic-soaked envelopes include standardized drug delivery, ease of use, and the avoidance of any potential interference with the regenerative properties of ECM caused by adding antibiotic directly into the material. The clinical availability of this novel antibiotic-eluting bioenvelope provides implanting physicians with a simple, reliable, and consistent means of delivering highly efficacious antibiotics directly to the surgical pocket, using an envelope that supports healthy wound healing, avoids fibrosis, and facilitates future reoperations. This latter quality of facilitating tissue integration and neovascularization of the device differentiates antibiotic-eluting bioenvelope from the non-biologic ABE, which, as demonstrated by the HEAL study, often promote fibrous encapsulation of the device.

## 6 Conclusions

Bioenvelopes for CIED implantation support healthy wound healing, effectively stabilize implanted devices, facilitate reoperation, and enhance the clearance of bacteria from the surgical pocket. Building on this platform, a next-generation antibiotic-eluting bioenvelope, now available for clinical use, elutes rifampin and minocycline directly into the surgical pocket. Preclinical studies indicate that this antibiotic-eluting bioenvelope is safe, elutes clinically meaningful levels of broad-spectrum antibiotics around the device, and eliminates pathogenic bacterial inoculates, both *in vitro* and *in vivo*. These bioenvelopes have consistently demonstrated support for healthy would healing, and emerging clinical evidence describes the benefits of bioenvelopes for improving ease of reoperation.

Additional clinical studies are required to establish the efficacy of the antibiotic-eluting bioenvelope for CIED implantations and to better define its role in therapy. Based on current evidence, the antibiotic-eluting bioenvelope may be considered for patients with high infection risk, thin or inadequate tissue for the surgical pocket, and those who are likely to undergo future reoperation.
